# Influence of Acid, Ethanol, and Anthocyanin Pigment on the Optical and Mechanical Properties of a Nanohybrid Dental Composite Resin

**DOI:** 10.3390/ma11071234

**Published:** 2018-07-18

**Authors:** Sukhyun Hwang, Shin Hye Chung, Jung-Tae Lee, Yong-Tae Kim, Yoo Jin Kim, Soram Oh, In-Sung Luke Yeo

**Affiliations:** 1 College of Medicine, Korea University (KU), Seoul 02841, Korea; illpd@naver.com; 2 Department of Dental Materials Science, Seoul National University School of Dentistry (SNUSD), Seoul 03080, Korea; den533@snu.ac.kr; 3 Department of Periodontology, Seoul National University School of Dentistry (SNUSD), Seoul 03080, Korea; jungtae1308@hanmail.net; 4 Department of Applied Statistics, Dankook University (DU), Yongin 16890, Korea; dataminer@empas.com; 5 Biological Science, Rice University (RU), Houston, TX 77005, USA; yk36@rice.edu; 6 Department of Conservative Dentistry, Kyung Hee University Dental Hospital (KHUDH), Seoul 02447, Korea; 7 Department of Prosthodontics, School of Dentistry and Dental Research Institute, Seoul National University (SNU), Seoul 03080, Korea

**Keywords:** color, composite resin, acidity, anthocyanin, ethanol

## Abstract

This study investigated the influences of acidity, ethanol, and pigment on the optical properties, microhardness, and surface roughness (R_a_) of a nanohybrid dental composite resin. A total of 108 disc-shaped specimens were fabricated using a nanohybrid dental composite and allocated into 36 different storage solutions according to the levels of pH (2.0, 3.0, 4.0, and 5.5), ethanol (0%, 20%, and 40%), and anthocyanin pigment (0%, 2.5%, and 12.5%). Measurements of the colorimetric parameter and the amount of color change (ΔE), translucency parameter (TP), microhardness, and surface roughness (R_a_) were performed at 24 h (baseline), 1-, 2-, 3-, and 4-weeks. Repeated measures of analysis of variance (ANOVA) with a Tukey honestly significant difference test and Pearson correlation analysis were carried out (α = 0.05). Pigment of 12.5% or 40% ethanol significantly increased the ΔE (*P* < 0.001, *P* = 0.048, respectively). Pigment of 2.5% or 12.5% significantly decreased the TP (*P* = 0.001, *P* < 0.001, respectively). Microhardness of composite resin stored in pH 2.0, 3.0, 4.0 solution was lower than that for pH 5.5 (*P* < 0.001). Pigment, ethanol, and pH did not influence the R_a_. TP change and ΔE, and R_a_ and ΔE had a significant positive correlation (*P* < 0.05). In conclusion, pigment and ethanol levels influenced the optical properties and acidity affected the microhardness of composite resin.

## 1. Introduction

Direct tooth restorations with dental composite resins constitute a significant proportion of dental practice due to an increasing demand for tooth-colored restorations [1]. The success of tooth-colored restorations depends on the adequate esthetics, shade match, and stability of restorations, as well as surface microhardness and compressive and flexural strength [2]. The staining of composite resin restorations is attributable to both external and internal factors. External causative factors include the production of colored components in plaque by chromogenic bacteria and the sorption of water-soluble stains from dietary constituents [3]. Internal causative factors include the rough surfaces of restorations and deterioration of the polymer network by water sorption [2]. 

A coupling agent is incorporated into the composite resin to interconnect the inorganic filler and organic matrix monomer [4]. Long-term storage in water induces decomposition of the silane coupling agent between the matrix and filler, which eventually caused water sorption by the resin matrix [5]. There have been studies on the development of new composite resins with an increased compatibility between the filler particle and matrix, including the silanization of silica particles [6]. Filtek Z350XT (3M ESPE, St. Paul, MN, USA) is a nanohybrid methacrylate-based composite resin which has a silane-treated nano-sized filler. Filtek Z350XT contains triethylene glycol dimethacrylate (TEGDMA) in its matrix, which is a diluent monomer used for controlling the high viscosity of other monomers. However, TEGDMA has been reported to present higher water sorption and water solubility [7,8]. According to Ferracane, composite resins are subject to both hygroscopic and hydrolytic effects that may influence their mechanical properties such as strength, wear resistance, and surface hardness [9].

Red wine has a higher risk of discoloration of dental composite restorations compared with tea, coke, orange juice, and sports drinks [10,11], yielding a clinically noticeable color change (ΔE > 3.3) [12]. The pigments within red wine are a main external causative factor in the discoloration of composite resin [10,11,13]. However, the level of alcohol and the acidity of red wine may affect the color stability of composite resin as internal causative factors. For example, a recent study reported the remarkable discoloration of composite resin by alcoholic beverages, such as beer and whiskey, which contained fewer pigments than red wine [14]. Alcohol-containing mouth rinses caused greater discoloration of composite resin than alcohol-free mouth rinses [15]. Methacrylate-based resins exhibit increased staining by coffee after 21 days of immersion in 75% ethanol, whereas immersion in phosphoric acid or citric acid does not potentiate staining by coffee [16]. Acidic sports drinks induce discoloration of composite resin [17,18], as well as a reduction in the surface microhardness [19]. Water sorption for some composite resins was increased after exposure to an acidic solution [20], which implies a possibility for stimulating degradation of the polymer network of methacrylate-based resin. Acidity, alcohols, and pigments complexly affect the mechanical and optical properties of composite resins [21]. However, it was very difficult to identify a study that evaluated the effects of these factors separately. To figure out whether these factors cause degradation of the dental composite at the outer layer, thereby affecting the mechanical properties, evaluation of the surface roughness and microhardness is required.

Tooth enamel possesses an inherent translucency; the property that permits the passage of the light but disperses the light so that objects cannot be seen clearly through this substance [22]. It is desirable for a composite resin to simulate and maintain the translucency of a natural tooth [23]. The translucency of a composite resin is primarily associated with the refractive indices of the resin matrix and fillers, which may change due to physicochemical reactions of the composite resins [24,25,26]. The translucency of methacrylate-based composite resins decreased after artificial aging [26] or following immersion in red wine [11,27].

The objective of the present study was to investigate the effect of one external factor (pigment) and two internal factors (ethanol, pH) on the color stability of a nanohybrid composite resin. Additionally, the effect of these factors on the change in translucency, microhardness, and surface roughness of the nanohybrid composite resin was investigated. The hypothesis underlying this investigation was that the pH of storage solutions and the concentrations of pigment and ethanol would affect the color stability, translucency, microhardness, and surface roughness of the nanofilled composite resin.

## 2. Materials and Methods

### 2.1. Specimen Preparation

In total, 108 disc-shaped specimens were fabricated with a methacrylate-based nanohybrid composite resin (Filtek Z350 XT) in a Dentin A2 shade. The composition of this composite resin is described in Table 1. The specimens were fabricated individually in a metallic mold containing a disc-shaped orifice (diameter 10 mm, height 1 mm). The composite resin was packed into the disc-shaped orifice in a single step, and a polyester strip was placed underneath and on the upper surface of the composite. A slide-glass was placed over the composite-mold assembly, and firm finger pressure was applied to compact the material. To prevent the formation of an oxygen inhibition layer, the composite resin increment was light-cured through the polyester strip. Each surface was cured for 40 s using a light-emitting diode light-curing unit (Elipar Freelight 2, 3M ESPE, St. Paul, MN, USA) with a minimum output of 1000 mW/cm^2^ at the tip. The composite-disc specimens were gently removed from the molds and stored in distilled water (DW) for 24 h at 37 °C. The specimens were divided into 36 experimental groups according to the amount of pigment (0.0%, 2.5%, and 12.5%), pH of the storage solution (pH 2.0, pH 3.0, pH 4.0, and pH 5.5), and concentration of ethanol (0%, 20%, and 40%). The modes of action of these three factors were divided into external causative factor (pigment) and intrinsic causative factors (pH, ethanol).

Three specimens were used in each group. The anthocyanin pigment and kuromanin chloride (Extrasynthese, Lyon, France) were added to DW to produce 2.5% and 12.5% solutions for the corresponding groups. Absolute ethanol (99.9%) was added to prepare storage solutions with 20% and 40% ethanol. Finally, the pH of the experimental solutions was adjusted by adding 99.7% acetic acid (Sigma-Aldrich, St. Louis, MO, USA) to the solutions.

Specimens were stored in a polyethylene tube containing DW in a dark incubator at 37 °C during the experiment. Three composite-disc specimens were immersed in each of the 36 storage solutions for 2 h per day for four weeks; the storage solutions were replenished for each immersion. At the end of four weeks, composite-disk specimens were air-dried. The measurements of colorimetric parameter, TP, microhardness, and surface roughness were performed at predetermined evaluation time points: 24 h (baseline) and 1-, 2-, 3-, and 4-weeks after specimen fabrication.

### 2.2. Colorimetric and Translucency Parameter

The color of the composite surface was measured using a spectrophotometer (Colorimeter, Model Tc-6Fx, Tokyo Denshoku Co., Tokyo, Japan). The absorption measurements were triplicated for each composite-disk specimen. For each color measurement, the values were expressed as CIE L*a*b* color space. L* is lightness, where 100 is white and 0 is black, and a* and b* are red–green and yellow–blue chromatic coordinates, respectively [28]. A positive a* value represents red, and a positive b* value represents yellow [28]. The light-source illumination corresponded to average daylight (D65). The colorimeter was calibrated before each measurement using the white calibrating sample supplied by the manufacturer. The color change (ΔE) of the composite-disk specimen for each period (1-, 2-, 3-, and 4-weeks) compared to the baseline (24 h) was calculated according to the following equation:

ΔE = {(ΔL*)^2^ + (Δa*)^2^ + (Δb*)^2^}^½^ (1)

where ΔL* = variation in lightness (black–white), Δa* = variation in a*-axis, and Δb* = variation in b*-axis in a distinct period. Values of ΔE greater than 3.3 were considered unacceptable, because 50% of the observers perceived the color difference when the ΔE was 3.3 [12].

The TP of each composite-disk specimen was calculated from color measurement with white and black backings using a colorimeter, as follows:

TP = {(L*_W_ − L*_B_)^2^ + (a*_W_ − a*_B_)^2^ + (b*_W_ − b*_B_)^2^}^½^ (2)

where the subscripts ‘W’ and ‘B’ refer to the CIE L*a*b values for each specimen against a white backing and a black backing, respectively [29,30].

### 2.3. Measurement of Microhardness

The surface microhardness of the nanohybrid composite was measured at the baseline and at 1-, 2-, 3-, and 4-weeks after storage using a microhardness tester (Fm 7, Future Tech Corp., Tokyo, Japan). The microhardness test was performed at a pressure of 500 g for 15 s at room temperature. The surface microhardness was measured on the bottom and top surface of the specimen and five measurements were recorded for each surface. The mean value was calculated and converted to Knoop hardness.

### 2.4. Surface Analysis

Surface roughness (R_a_) was quantitatively measured using confocal laser scanning microscopy (CLSM; LMS 5-Pascal, Carl Zeiss, Oberhausen, Germany) at the baseline and at 1-, 2-, 3-, and 4-weeks after immersion in the experimental solutions. Three measurements were performed on each specimen for each evaluation period, and the mean value was calculated. A low-pass Gaussian filter was used to calculate the three-dimensional roughness parameters, R_a_. Arithmetic mean deviation of the peak-to-valley height of the two-dimensional surface was used for statistical analysis.

### 2.5. Statistical Analysis

Repeated measures of analysis of variance (ANOVA) with a Tukey honestly significant difference test were performed to determine whether the ΔE [TP, microhardness, R_a_] changes significantly according to different concentrations of pigment [concentration of ethanol, pH], and whether the ΔE [TP, microhardness, R_a_] changes significantly according to storage period (1-, 2-, 3-, and 4-weeks). Pearson’s correlation analysis was performed to examine the relationship among ΔE, TP change, microhardness, and R_a_ at 1-, 2-, 3-, and 4-weeks, respectively. Statistical analysis was carried out using the SPSS software (SPSS version 22; IBM, Armonk, NY, USA). The level of statistical significance was established at *P* < 0.05.

## 3. Results

### 3.1. Colorimetric and Translucency Parameter

**Figure 1:**
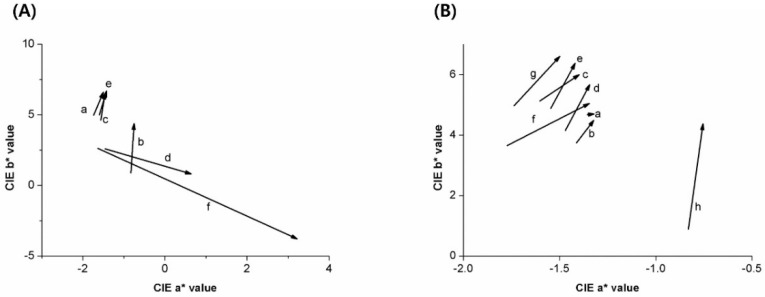
Mean a* and b* values changed from one week to four weeks (arrowhead) according to concentrations of ethanol and pigment (**A**), according to pH and concentration of pigment (**B**). (**A**) pH 5.5 solutions with ethanol 0 and pigment 0 (**a**); ethanol 0 and pigment 12.5% (**b**); ethanol 20% and pigment 0 (**c**); ethanol 20% and pigment 12.5% (**d**); ethanol 40% and pigment 0 (**e**); ethanol 40% and pigment 12.5% (**f**). Without pigment, storage in different ethanol concentrations (**a**,**c**,**e**) demonstrated similar patterns to storage solution with pigment and no ethanol (**b**), the color changed to increased redness and yellowness. However, color changed towards redness and blueness when both pigment and ethanol was used (**d**,**f**). (**B**) Non-ethanol solutions with pH 2 and pigment 0 (**a**); pH 2 and pigment 12.5% (**b**); pH 3 and pigment 0 (**c**); pH 3 and pigment 12.5% (**d**); pH 4 and pigment 0 (**e**); pH 4 and pigment 12.5% (**f**); pH 5.5 and pigment 0 (**g**); pH 5.5 and pigment 12.5% (**h**). At the same pH, the color change, which was towards increased redness and yellowness, was more severe when the pigment was added (**a**–**h**).

**Figure 2:**
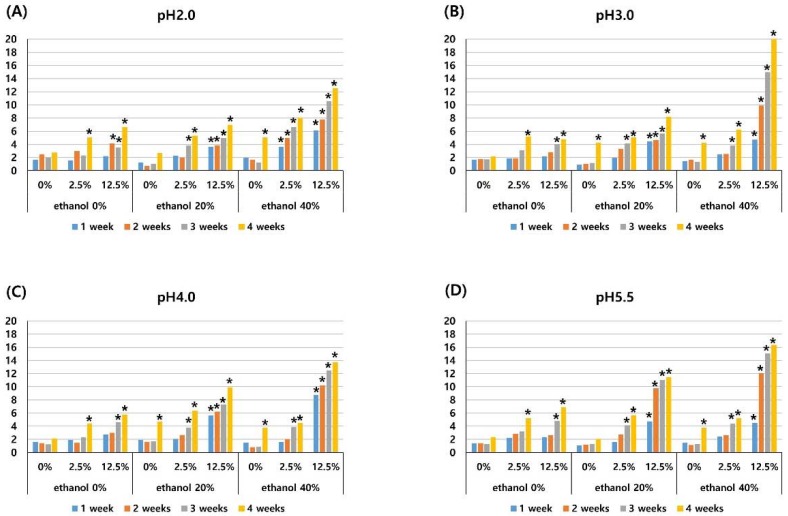
Mean color changes (ΔE) of the composite resin after different periods of storage (1-, 2-, 3-, and 4-weeks) in media of pH 2.0 (**A**), pH 3.0 (**B**), pH 4.0 (**C**), pH 5.5 (**D**) with three different pigment concentrations (0%, 2.5%, 12.5%), and three different ethanol concentrations (0%, 20%, 40%). An asterisk (*) represents a clinically noticeable color change (ΔE > 3.3).

Overall, L value (lightness) was decreased, a* value was increased (towards redness), and b* value was decreased (towards blueness) as the concentration of the pigment was increased. The amount of change in a* and b* values of representative specimens at 1-, 2-, 3-, and 4-weeks is shown in Figure 1. Without the pigment, storage in different ethanol concentrations demonstrated similar patterns for the color change at pH 5.5, as shown in Figure 1(Aa,c,e). When the pigment was added without ethanol a* value increased (towards redness) and b* value increased (towards yellowness) as time progressed, as shown in Figure 1(Ab). Whereas, when the pigment and ethanol were combined a* value increased (towards redness) and b* value decreased (towards blueness) as time progressed, as shown in Figure 1(Ad,f). When exposed to a solution without ethanol, a* value increased (towards redness) and b* value increased (towards yellowness) as time progressed, as shown in Figure 2B. The higher pigment yielded an increased color change with a higher a* value and lower b* value compared with each counterpart of the same pH, shown in Figure 2(Ba–h).

The color change (ΔEs) of the specimens increased significantly at each time of measurement (1-, 2-, 3-, and 4-weeks) (*P* < 0.001). There was no significant difference between ΔE in a storage solution with 0% pigment and that in a storage solution with 2.5% pigment (*P* = 0.131), whereas 12.5% pigment significantly increased the ΔE when compared with a solution of 0% or 2.5% pigment (*P* < 0.001), as shown in Table 2. Storage in a 40% ethanol solution presented significantly greater ΔE than that of a solution with 0% ethanol (*P* = 0.048), while there was no significant difference between ΔE in a storage solution with 20% ethanol and 0% ethanol. ΔE did not change significantly according to the pH of the solution, as shown in Table 2. The mean color changes (ΔE) in each experimental solution at each experimental period are shown in Figure 2. Specimens which had a clinically noticeable color change (ΔE > 3.3) were indicated with an asterisk. When the level of pigment was 2.5% and 12.5%, a noticeable color change was induced, as shown in Figure 2. If the concentration of ethanol was 40%, the color change was clinically noticeable even if the pigment was not used, as shown in Figure 2. When the ethanol concentration was 20% without pigment for a pH of 3.0 or 4.0, there was a clinically noticeable color change, as shown in Figure 2B,C.

**Figure 3:**
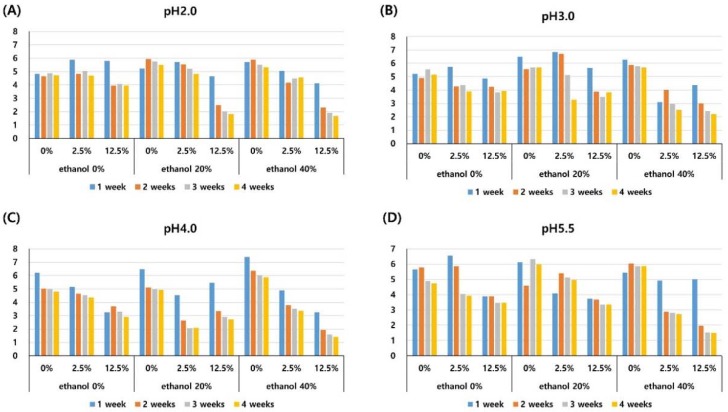
Mean translucency parameters of the composite resin after different periods of storage (1-, 2-, 3-, and 4-weeks) in media of pH 2.0 (**A**), pH 3.0 (**B**), pH 4.0 (**C**), pH 5.5 (**D**) with three different pigment concentrations (0%, 2.5%, 12.5%), and three different ethanol concentrations (0%, 20%, 40%).

The translucency parameter (TP) decreased as the storage period increased, and this change was significant at each time of measurement (1-, 2-, 3-, and 4-weeks) (*P* < 0.001). A storage solution with 2.5% pigment significantly decreased the TP compared to a solution with 0% pigment (*P* = 0.001). The solution with 12.5% pigment significantly decreased the TP when compared with both the solution with 0% pigment (*P* < 0.001) and the solution with 2.5% pigment (*P* = 0.002), as shown in Table 2. The TP did not change significantly according to the pH or ethanol concentration of the solution. The mean TP in each experimental solution is presented in Figure 3. 

### 3.2. Measurement of Microhardness

**Figure 4:**
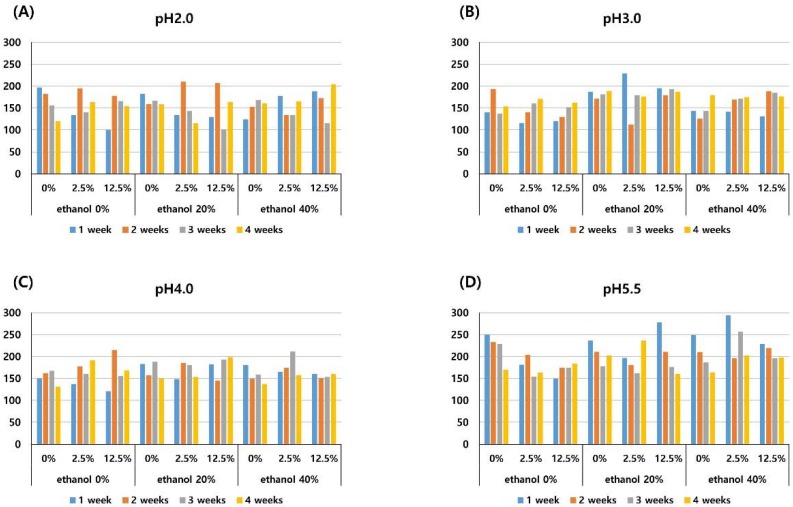
Mean microhardness values of the composite resin after different periods of storage (1-, 2-, 3-, and 4-weeks) in media of pH 2.0 (**A**), pH 3.0 (**B**), pH 4.0 (**C**), pH 5.5 (**D**) with three different pigment concentrations (0%, 2.5%, 12.5%), and three different ethanol concentrations (0%, 20%, 40%).

The mean microhardness of the composite resin in each experimental solution is presented in Figure 4. The microhardness of composite resin stored in the pH 5.5 solution was significantly higher than those stored in solutions of pH 2.0, 3.0, and 4.0 (*P* < 0.001), as shown in Table 2. The pigment and ethanol concentrations did not significantly affect the microhardness. The microhardness did not significantly change according to the length of the storage period (*P* > 0.05), as shown in Figure 4.

### 3.3. Surface Analysis (R_a_)

**Figure 5:**
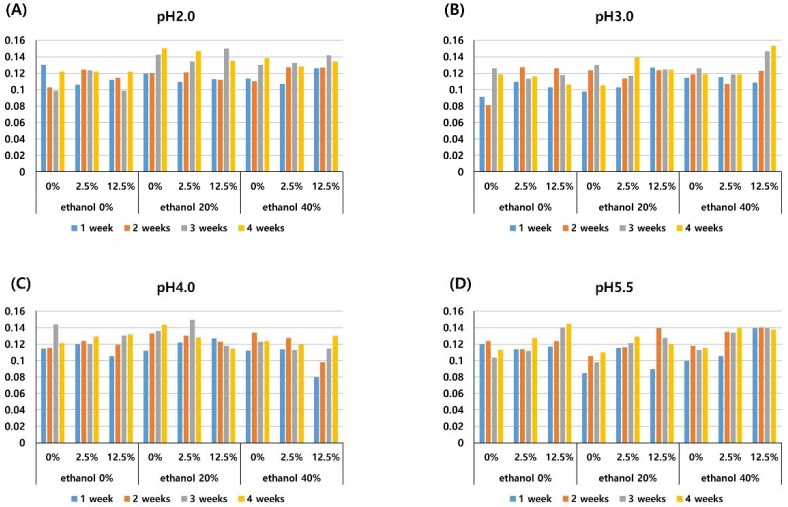
Mean surface roughness values (R_a_) of the composite resin after different periods of storage (1-, 2-, 3-, and 4-weeks) in media of pH 2.0 (**A**), pH 3.0 (**B**), pH 4.0 (**C**), pH 5.5 (**D**) with three different pigment concentrations (0%, 2.5%, 12.5%), and three different ethanol concentrations (0%, 20%, 40%).

The average R_a_ values of the specimens in each experimental solution are presented in Figure 5. The R_a_ was not significantly different among solutions of different pigment concentration, ethanol concentration, or pH. The R_a_ increased as the storage period increased, and this change was significant at each time of measurement (1-, 2-, 3-, and 4-weeks) (*P* < 0.001), as shown in Figure 5.

### 3.4. Correlation between ΔE, TP Change, Microhardness, and R_a_

Color change (ΔE) and TP change showed a significant positive correlation at 1-, 2-, 3-, and 4-weeks (Pearson r = 0.508, 0.701, 0.774, 0.717 at 1-, 2-, 3-, and 4-weeks, respectively; *P* = 0.002 at 1-week, *P* < 0.001 at 2-, 3-, and 4-week.) Color change (ΔE) and R_a_ showed a significant positive correlation at 3- and 4-weeks (Pearson r = 0.35, 0.383, respectively at 3- and 4-weeks; *P* = 0.036, 0.021, respectively.) There was no significant correlation between ΔE and microhardness; TP change and microhardness; TP change and R_a_; microhardness and R_a_.

## 4. Discussion

### 4.1. Colorimetric and Translucency Parameter

In the present study, with higher concentrations of the pigment, a decreased lightness and a color change to redness and blueness were observed while some previous studies have reported increased b* values (towards yellowness) after immersion in red wine [14,31]. These previous studies used commercial wines; therefore, it is possible that other components or pigments in red wine contributed to the increased b* value (towards yellowness).

In this study, the nanohybrid composite resin showed distinct color changes that depended on the levels of pigment (anthocyanin) and on the alcohol content of the storage media. Previous studies have suggested that the alcohol content of wine facilitated staining by softening the resin matrix [13,32]. However, assessments of the effects of alcohol itself, on the optical properties of nanohybrid composite resin have not been performed. In the present study, immersion in 40% ethanol without pigment caused clinically detectable discoloration of the nanohybrid composite after four weeks of storage, as shown in Figure 2. Previous studies demonstrated that the ethanol-containing solution was readily absorbed by the resin monomer of the nanohybrid composite, i.e., bisphenol glycidyl methacrylate (bis-GMA), ethoxylated bisphenol-A dimethacrylate (bis-EMA), urethane dimethacrylate (UDMA), and TEGDMA [31,33]. As the nanohybrid composite resin used in this study (Filtek Z350XT) contains bis-GMA, bis-EMA, UDMA, and TEGDMA, it is likely that ethanol was easily absorbed by the resin matrix.

The color change caused by anthocyanin was potentiated by the presence of ethanol by increasing the penetration of the pigment into the resin. In the solution of pH 3.0, 4.0, 5.5, without ethanol, 2.5% anthocyanin caused a clinically detectable color change (ΔE > 3.3) after four weeks of storage, and 12.5% anthocyanin caused a clinically detectable color change after 3 weeks of storage, as shown in Figure 2. Whereas, with 20% and 40% ethanol, 2.5% anthocyanin caused a clinically detectable color change after three weeks of storage, and 12.5% anthocyanin caused a clinically detectable color change after one week of storage, as shown in Figure 2. The results of the present study are in accordance with that of the study by Benetti et al., who reported an increased staining susceptibility of Filtek Z350XT after storage in 75% ethanol for three weeks [16]. 

The pH of the solution did not significantly affect the color change of the composite resin, as shown in Table 2 and Figure 2. When the ethanol concentration was 20% without pigment at a pH of 3.0 or a pH of 4.0, there was a clinically noticeable color change, shown in Figure 2B and 2C, while there was no clinically noticeable color change in the pH 2.0 and pH 5.5 solution, as shown in Figure 2A and 2D. It is assumed that the degeneration by ethanol was accelerated by the acidic solution of pH 3.0–4.0.

The TP decreased irrespective of the storage solution. This finding is in agreement with those of previous studies which have reported the decreased translucency of methacrylate-based nanohybrid resin stored in water [27,31]. The decrease in TP was dependent on the concentration of anthocyanin pigment in this study, i.e., higher concentrations of pigment induced a greater reduction in TP at identical pH and ethanol concentrations, see Table 2 and Figure 3. As the resin matrix absorbed water, the penetration of the anthocyanin pigment into the resin matrix was enhanced and thereby decreased the light transmittance and translucency. 

The specimens immersed in the pH 5.5 solutions without ethanol or pigment demonstrated decreased TPs and slight color changes (mean ΔE = 2.32 at four weeks). These findings are in agreement with those of previous studies that have reported decreased translucency and slight color changes of Filtek Z350XT immersed in water [27,31,34]. The discoloration may result from the aging of the composite. According to Ferracane and Curtis et al., the storage of the composite resin in water lead to its degradation [9,35].

### 4.2. Microhardness and Surface Roughness

In this study, a low pH (pH 2.0, 3.0, 4.0) induced a reduction of surface microhardness, see Table 2 and Figure 4, which is consistent with the study of Erdemir et al. which presented decreased microhardness of composite resin by acidic drinks [19]. Erdemir et al. reported that composite resin exposed for one month demonstrated a lower microhardness compared with that which had undergone one week of exposure. On the contrary, in the present study, there was no significant difference among 1-, 2-, 3-, and 4-week measurements. It is probably due to this study being conducted on different composite resins.

Neither the pigment, ethanol, or pH had a significant influence on the R_a_, as shown in Table 2. The R_a_ was dependent on the storage period. The R_a_ increased irrespective of the storage solution the longer the specimen was stored in the experimental solution, as shown in Figure 5. Meanwhile, Benetti et al. demonstrated no change in the roughness of composite resins which were immersed in water or ethanol for up to 180 days [16]. Unlike Benetti et al., in the present study, polishing of light-cured composite resin specimen was not performed, which might cause increased roughness as the storage period extended.

### 4.3. Correlation between ΔE, TP Change, Microhardness, and R_a_

The color change (ΔE) and TP change had a strong positive correlation (Pearson r > 0.5). The higher the ΔE, the more the TP changed, because a storage solution of 12.5% pigment led to a significant effect on both the color change and TP change. Color change (ΔE) and R_a_ showed a significant positive correlation at 3- and 4-weeks. It is speculated that increased surface roughness contributed to accelerated discoloration. A previous study suggested that surface irregularities influenced the discoloration of composite resins [36].

### 4.4. Experimental Design

According to Miyazaki et al., heating the direct composite resin to 170 °C after light curing increased the flexural strength compared to that when only light curing alone was performed [37]. Conversely, Magnet et al. reported that heating the light-cured direct composite resin did not enhance the micro-tensile bond strength [38]. In this study, the composite resin specimens were stored in the experimental solution at body temperature in order to replicate the actual situation of drinking.

The acidity of red wine affects fermentation and maturation, prevents microbial growth and spoilage, and is important for its storage. A pH between 3.2 and 3.6 is recommended as suitable for the fermentation of red wine, and a pH range of 3.2 to 3.3 is suitable for its storage [39]. Therefore, pH values of 2.0, 3.0, 4.0, and 5.5 were examined in this study.

In the present study, polishing of the light-cured specimen was not performed. A polyester-film-covered surface produced the smoothest surface [40]. Patel et al. noted that the surface beneath the polyester film strip had a lower degree of polymerization and was consequently more susceptible to discoloration [32]. Nevertheless, to investigate the effects of acidity, ethanol, and pigment on the surface roughness, polishing was not used in the present study because manual polishing alters the surface profile.

As color stability varies with the composition of the resin matrix and the type of filler [10,11,17,18,31], extrapolation of the results of the present study to general composite resin is somewhat unreasonable. The optical characteristics of a composite resin depend on its light absorption and scattering properties [41,42]. Absorption is affected by the organic matrix, whereas scattering depends on the mismatch between the refractive index of the organic matrix and filler particles in addition to filler size, distribution, and load [42,43]. Marjanovic et al. demonstrated that the color change depended on the shade of the composite resin [44]. According to Haas et al., the type and amount of opacifier in the composite resin such as titanium oxide (TiO_2_), aluminum oxide (Al_2_O_3_), and zirconium oxide (ZrO_2_) influenced the translucency [45]. Therefore, further investigations on the change of color and the translucency of composite resins with different compositions and shades are needed.

Most red wines contain 12% ethanol [46]. Further studies concerning lower concentrations of ethanol, and different types of composite resin are required. Another limitation of the present study is the lack of identification of the mechanism by which the colorimetric parameter, translucency, microhardness, and surface roughness had changed. Such mechanisms were beyond the scope of our research and will be clarified in future studies.

## 5. Conclusions

Anthocyanin pigment, the external causative factor, had a significant influence on the color change of the nanohybrid composite resin at a concentration of 12.5%, and on the translucency parameter at concentrations of 2.5% or 12.5%. Ethanol content, rather than the acidity of the solution, is a more critical intrinsic factor for the alterations in color of the nanohybrid composite resin. Exposure of the nanohybrid composite to a solution with pH 4.0 or less adversely affected the microhardness.

## Figures and Tables

**Table 1:** The composition of nanohybrid composite (Filtek Z350XT) used in this study.

Filler (63.3 Volume %)	Matrix
silica nanoparticles (5–20 nm) zirconia/silica clusters (0.6–1.4 μm)	Bis-GMA, Bis-EMA, UDMA, PEGDMA, TEGDMA

Bis-GMA: bisphenol glycidyl methacrylate; bis-EMA: ethoxylated bisphenol-A dimethacrylate; UDMA: urethane dimethacrylate; PEGDMA: polyethylene glycol dimethacrylate; TEGDMA: triethylene glycol dimethacrylate.

**Table 2:** P-values from repeated measures of analysis of variance (ANOVA) carried out according to one of three factors (pigment concentration, ethanol concentration, or pH of storage solution) and time (1-, 2-, 3-, and 4-weeks). N.B.: There was no statistical significance (*P* > 0.05).

FACTOR	PAIRWISE COMPARISON (VS)		ΔE	TP CHANGE	MICRO-HARDNESS	RA
PIGMENT (%)	0	2.5	N.S.	0.001	N.S.	N.S.
	0	12.5	<0.001	<0.001	N.S.	N.S.
	2.5	12.5	<0.001	0.002	N.S.	N.S.
ETHANOL (%)	0	20	N.S.	N.S.	N.S.	N.S.
	0	40	0.048	N.S.	N.S.	N.S.
	20	40	N.S.	N.S.	N.S.	N.S.
PH	5.5	4.0	N.S.	N.S.	<0.001	N.S.
	5.5	3.0	N.S.	N.S.	<0.001	N.S.
	5.5	2.0	N.S.	N.S.	<0.001	N.S.
	4.0	3.0	N.S.	N.S.	N.S.	N.S.
	4.0	2.0	N.S.	N.S.	N.S.	N.S.
	3.0	2.0	N.S.	N.S.	N.S.	N.S.
